# Stability of Lifestyle Behavior – The Answer to Successful Cognitive Aging? A Comparison of Nuns, Monks, Master Athletes and Non-active Older Adults

**DOI:** 10.3389/fpsyg.2019.01347

**Published:** 2019-06-07

**Authors:** Nadja Schott, Katja Krull

**Affiliations:** Department of Sport and Exercise Science, University of Stuttgart, Stuttgart, Germany

**Keywords:** master athletes, working memory, attention and inhibitory control, lifestyle engagement, older adults

## Abstract

**Background:** Epidemiological studies of the effect of physical activity on cognition demonstrated an inverse relationship between physical activity and cognitive decline. However, such health behaviors are hardly invariable over time. The relative homogeneity of the adult lifestyle of nuns/monks as well as master athletes reduces the likelihood of confounding due to differences in their participation in regular life-long physical activities. The purpose of this study was to determine if there were differences in cognitive functions between nuns/monks, master athletes and sedentary, but otherwise healthy older adults. Additionally, we examined associations between demographic variables (education, sex, age), BMI, physical activity, exercise, and fitness and cognitive performance.

**Methods:** We recruited three groups of healthy participants without cognitive deficits: (1) Nuns/Monks (*n* = 20; age 77.5 ± 5.56; 5 M, 15 W), (2) Master Athletes (*n* = 20; age 76.5 ± 5.33; 12 M, 8 W), and (3) Sedentary (*n* = 20; 76.4 ± 5.96, 6 M, 14 W). Cognitive performance (working memory, inhibition) was measured with a n-back task and a flanker task, participation in physical activities with the “German-PAQ-50+,” and physical fitness with the 30s chair stand and arm curl test.

**Results:** As predicted, ANOVA comparing groups revealed the three groups differed in cognition, physical activity, and physical fitness with inactive older adults performing lower on all tests than the other two groups. Hierarchical regression analyses showed a positive influence of lifestyle stability on accuracy and reaction time for working memory and inhibitory performance. The highest correlation coefficients for fitness and cognitive performance emerged for the group of nuns and monks.

**Conclusion:** Life-long stability of an active lifestyle may confer benefits to some aspects of working memory, attention, and inhibitory control. Longitudinal studies are recommended to further examine the causal relationship of lifestyle stability and cognitive function in such specific cohorts.

## Introduction

Worldwide we find an increasing number of individuals becoming centenarians and live “sharp as a tack” and/or physically fit while many others suffer varying degrees of cognitive and motor deterioration with increased morbidity, dependence, and mortality (Negash et al., 2013). Having a low risk of disease and disease-related disability, high cognitive, and physical functional capacity, and being actively engaged with life have been agreed upon as essential components of successful aging (Rowe and Kahn, 1997). Studies have also shown that healthy lifestyles are even as influential as genetic factors in helping older adults to attenuate the age-related limitations (Rea, 2017). Common behaviors contributing to healthy aging are eating in moderation, sufficient hydration, exercise, purposeful living (e.g., life philosophy, volunteerism, “hard work”), spirituality, and maintaining social support systems (Pignolo, 2018).

Physical activity, exercise and fitness^1^ are key factors in maintaining or even improving cognitive performance. A number of studies have found that high physical activity levels or better low levels of sedentariness (prolonged and uninterrupted periods of sitting) as well as high aerobic capacity are associated with neuroprotective effects on structural and functional brain health (e.g., increased gray and white matter of the prefrontal and temporal cortices, neurogenesis, synaptogenesis) in healthy older adults as well as patients with Mild Cognitive Disorder or Dementia (for a recent review see Tyndall et al., 2018). Despite numerous studies in humans and animals, the mechanisms for the effect of exercise on cognitive control are unclear. However, some aspects of the process, such as increased astrocyte glycogen storage, increased expression of BDNF, PGC-1α signaling and altered IL-6 production of the skeletal muscle, seem to mediate the benefits of exercise for cognitive function (Norman et al., 2018).

Studies examining the relationship between physical activity, exercise, fitness and cognition usually included individuals who were not particularly inclined to physical activity and exercise or were shown to rather exercise irregularly. Despite the knowledge of healthy lifestyle components only ∼36% of older adults in the United States (Keadle et al., 2016) and ∼58% in Europe (Marques et al., 2015) meet the physical activity guidelines of 150 min/week of moderate physical activity and/or 75 min/week of physical activity. Master athletes (MA; exercise on a regular basis to compete in organized competitive sport) on the other hand, maintain high levels of systematic exercise and retain better physical function, muscular strength and body fat levels than age-matched non-athletes (McKendry et al., 2018). They have been proposed as an excellent model for the best attainable trajectory of aging (Geard et al., 2017; Lazarus and Harridge, 2017). Only few studies have examined brain function and cognitive performance in MA compared to the typically more or less active older adult (Taran et al., 2013; Thomas et al., 2013; Tseng et al., 2013; Alfini et al., 2016; Zhao et al., 2016; Wouters et al., 2017; Dupuy et al., 2018). MA demonstrated greater resting cerebral blood flow in the posterior cingulate cortex (Thomas et al., 2013), higher gray and white matter concentrations in the right parietal and occipital lobes (Tseng et al., 2013); better executive control in a dual-task (Dupuy et al., 2018), but no relationship between physical activity and working memory, executive function, and visuospatial short-term memory (Wouters et al., 2017).

In addition to physical activity, other healthy behaviors such as religious (beliefs and practices related to the Transcendent) and/or spiritual (intrinsic part of being human; comprises a sense of connectedness to others) involvement as well as mindfulness meditation (awareness that arises through paying attention, on purpose, in the present moment, nonjudgmentally) influences various body-mind mechanisms (Koenig, 2012; Baer, 2019). A growing body of research shows that these forms of healthy behaviors reduce stress, depression, pain and anxiety, improves attention and memory, and promotes self-regulation as well as empathy (Malinowski et al., 2015; Geiger et al., 2016; Gonçalves et al., 2017). That includes evidence suggesting that different forms of religious and spiritual behavior as well as mindfulness meditation may protect the brain from normal cortical thinning (a sign of cognitive aging) and improve cognitive performance in elderly people (Giordano and Engebretson, 2006; Miller et al., 2014; Muehsam et al., 2017). Two iconic studies that have been key to advancing our knowledge of changes in motor and cognitive performance are the Religious Order Study and the Nun Study (Snowdon, 2003; Wilson et al., 2012; Latimer et al., 2017; Bennett et al., 2018). The Religious Order Study comprises *n* = 1240 Catholic nuns, priests, and brothers (Wilson et al., 2012), while the Nun Study comprises *n* = 678 Roman Catholic School Sisters of Notre Dame (Latimer et al., 2017). Using a time-varying effects model, Boyle et al. (2017) showed for data from the Religious Orders Study and the Rush Memory and Aging Project that a global motor measure declined with a mean 0.024-unit per year until a mean of 2.46 years before death when rate of decline increased nearly fivefold to -0.117-unit per year. Furthermore, the authors described that a global cognitive measure declined with a mean of 0.027-unit per year until a mean of 2.76 years before death when rate of decline increased more than 13-fold to -0.371-unit per year (Boyle et al., 2017). In comparison with the Nun Study, healthy older adults from the Honolulu-Asia Aging Study were found to have differences in prevalence of Alzheimer disease neuropathologic change, small vessel vascular brain injury, and Lewy body disease (Latimer et al., 2017). The authors suggest that lifestyle factors are responsible for their results in addition to gender and ethnicity.

Such health lifestyle behaviors are hardly invariable over time, adherence to a constant healthier lifestyle is moderate at best (Petersen et al., 2015). The relative homogeneity of the adult lifestyle of nuns/monks as well as master athletes reduces the likelihood of confounding due to differences in their participation in regular life-long physical activities. For example, due to their specific living conditions, members of religious orders are much more homogeneous than any other population group with regard to many health-relevant aspects. Nuns and monks maintain a lifestyle determined by vows with an almost identically regulated daily routine regarding sleep rhythm, working hours, physical and mental activities as well as phases of rest. In addition, all members of the order have almost identical living conditions, a comparable diet and the same access to medical care. In comparison to the typical population, nuns and monks cultivate a life largely free of social stress factors. They do not have to provide for themselves or a family and do not suffer from marital problems and financial burdens or worries in connection with child-raising and individual old-age provision. Results from the German-Austrian Cloister-Study show that 33.1% of the nuns and monks surveyed are active in sports more than once a week; a further 36.8% state that they carry out relaxation and meditation exercises every day; 54.7% describe their health status as excellent, and 20.8% often feel nervous or stressed (Wiedemann et al., 2014).

Several hypotheses and models have been advanced to account for a healthy lifestyle-induced cognitive protection (e.g., through exercise, well-balanced diet, minimized psychosocial stress): “brain reserve capacity” (Satz, 1993), “cognitive reserve capacity” (Stern, 2002), “neurocognitive scaffolding” (Reuter-Lorenz and Park, 2014), and recently the “Adaptive Capacity Model” (Raichlen and Alexander, 2017). The brain reserve capacity is defined as the brain’s resilience to pathological damage, for example, a lesion might lead to impairment in one individual with a low brain reserve capacity, but not in another individual with a higher brain reserve capacity (Christie et al., 2017). Cognitive reserve capacity refers to the attempt of the brain to compensate and to adapt following the onset of brain pathology (for a recent discussion see Nilson and Lövdén, 2018). However, both concepts are described as still hypothetical due to the only indirect measures of its operation (see for an extended discussion Dixon and Lachman, 2019). The Scaffolding Theory of Aging and Cognition (STAC) is a conceptual, dynamic model that attempts to explain different cognitive performance levels, which are caused by individual adverse and compensatory neuronal processes. Adverse age effects (neuronal degradation) are based on neuronal challenges (structural changes such as atrophy) and functional degradation (e.g., reduced specificity). They determine the cognitive functional level in interaction with advantageous processes (compensatory scaffolding), which try to counteract degradation. This includes plasticity, for example by recruiting additional neuronal networks (Park and Reuter-Lorenz, 2009). The original model was extended by a revised version (STAC-r), which considers two additional influencing factors. The compensatory scaffold can be improved by interventions such as a healthy lifestyle (accumulation of neuronal resources) or worsened by negative influences such as stress or the APOE 𝜖4 allele (degradation of neuronal resources) (Reuter-Lorenz and Park, 2014). Lifestyle factors play not only a role in the STAC-r model, but also in the Adaptive Capacity Model. Raichlen and Alexander (2017) proposed the Adaptive Capacity Model explaining the positive benefits of cognitively and physically demanding exercise: The brain responds to the combination of aerobic activity with control of motor systems, spatial navigation and memory, executive functions, and the control of sensory and attentional systems with a neuroplastic adaptive response by increasing capacity to lower energy costs. However, periods of inactivity lead to decreased structure and associated function, as reflected by age-related regional brain atrophy (Raichlen and Alexander, 2017). Conversely, no evidence can be found for a cognitive benefit (attention, memory, perception, executive functions, cognitive inhibition, cognitive speed, and motor function) of improved cardiorespiratory fitness in healthy older adults (Young et al., 2015). Only when combined with challenging cognitive exercise (e.g., dual task training) can positive effects on cognitive performance be observed (Gheysen et al., 2018). Lövdén et al. (2017, p. 473) categorize these frameworks and theoretical concepts explaining the influence of lifestyle factors in three dimensions: (a) through improving brain functioning and performance in younger age without altering brain aging *per se*, (b) by aiding compensatory reactions to primary brain aging, and (c) by fostering maintenance of a young–adults like brain in old age. However, the authors also state that we need to discover and measure what matters focusing on between-person differences in change.

Typically, scientists in the fields of cognitive aging, mild cognitive impairment and dementia focus on the description and explanation of age- and disease-related cognitive losses and the transition to dementia. However, a promising new approach focuses on the study of relatively healthy “optimal,” “successful” or “exceptional” “high-performing older adults” and the question of which risk and protective factors are present in these physically and/or cognitively exceptional individuals (Gefen et al., 2014; Borelli et al., 2018). SuperAgers are defined as individuals aged 80 years or older with memory ability similar or superior to control participants 20–30 years younger. They show no signs of cortical atrophy relative to control participants (Harrison et al., 2012), their anterior cingulate cortex is significantly thicker (Gefen et al., 2014; Sun et al., 2016), and they show higher levels of physical activity (Bott et al., 2017). A recent study, examining different cognitive domains (memory, attention, language, visuospatial abilities) and lifestyle factors (physical and social activities, cognitively demanding activities, sleep, smoking, alcohol, stress) resulted in counter-intuitive results: superior cognition in older adults was associated with a busier and more stressful as well as socially isolated lifestyle in their midlives (Yu et al., 2019). The authors argue that longer exposure to physically and cognitively stimulating activities with a moderate stress level may serve to build up a larger capacity for neurocognitive scaffolding. On the other hand, the adherence to a higher number of lifestyle factors is associated with better cognitive function in later life (Clare et al., 2017; Weng et al., 2018). However, these lifestyle factors are typically measured at a single point in time, assessed via subjective self-reporting ratings, and tend to change over time (Mulder et al., 1998; Picavet et al., 2011). Overall, it is unclear if additional benefits of physical activity, fitness or meditation emerge from continued practice.

To our knowledge, no study has examined the effects of stable healthy lifestyle profiles on cognitive and motor performance. Therefore, the primary aim of this study was to determine whether there were differences in cognitive functions (working memory, inhibition, cognitive flexibility) between nuns/monks, master athletes and sedentary, but otherwise healthy older adults. Given the broad differences in lifestyle factors – such as BMI, physical activity, exercise, and fitness – of these three groups, we hypothesized that lifestyle stability in Master Athletes and nuns and monks will result in better cognitive performance. Additionally, we examined associations between demographic variables (education, sex, age), BMI, physical activity, exercise, and fitness and cognitive performance. We predicted stronger associations between fitness (as an expression of current exercise) and cognition than physical activity and exercise and cognition (Wouters et al., 2017).

## Materials and Methods

### Study Participants

Sixty healthy older adults (23 men, 37 women) with no signs of dementia selected by the Montreal Cognitive Assessment (MoCA; score > 25; Nasreddine et al., 2005) were recruited from the southern part of Germany. The participants were divided into three groups reflecting different types and stability of lifestyle behaviors: (1) Nuns/Monks (NM; *n* = 20; 69–90 years of age), (2) Master Athletes (MA; *n* = 20; 69–90 years of age), and (3) community-dwelling older adults (CD; *n* = 20; 68–91 years of age).

The nuns and monks lived in three different cloisters in the south of Germany (men: *n*_total_ ∼30, *n*_≥__65__years_ ∼52%; women: *n*_total_ ∼90, *n*_≥__65__years_ ∼60%), but – relative to the general population – order members are a homogenous population concerning a number of individual characteristics possibly relevant for health such as diet, activities of daily living, religiosity, and living arrangements (see also the German-Austria Cloister Study; Wiedemann et al., 2014). The master athletes in this study participated regularly in track and field competitions at European and World Master Athletics Championships, and were recruited through the German Track and Field Association (dlv). They exercise on average 4 h per week, had a track and field experience of at least 20 years and reported a balanced diet, and healthy sleep patterns. The group of community-dwelling older adults was mentally active (e.g., participation in hobbies such as craft, solving puzzles, playing an instrument, volunteering), but participated in little or no structured exercise. They were recruited through community newsletters and meetings places for older adults.

Inclusion criteria were age 65 years or more with normal or corrected-to-normal vision, and able to walk with or without any assistive device. Furthermore, individuals were screened for eligibility to participate based on their medical history. They were excluded if they were medically unstable based on self-report of the following: cardiopulmonary disease with symptoms such as shortness of breath or chest pain that would limit participation; current infectious, inflammatory or terminal conditions; prior neurological disease such as stroke or Parkinson’s disease; acute illness, injury or pain at the time of testing that would limit testing performance. All participants were fluent German speakers.

All participants were informed of the nature and aim of the study, and signed a consent form. All procedures were in accordance to the Declaration of Helsinki with ethical standards, legal requirements and international norms. An internal ethics committee at the University of Stuttgart approved the study.

### Measures

#### Cognitive Assessment

##### N-back task

We used the N-back task introduced by Yun et al. (2010). All tasks were designed and presented by E-prime 2.0^2^ (Psychology Software Tools, United States) on a 17′′ computer screen with a resolution of 1.920 × 1200 pixels. Participants had a distance of ∼70 cm from the screen, remained at this distance during the trials, and completed the n-back tasks, starting with a 0-back task followed by the 1-back and 2-back tasks in consecutive order.

**Figure 1:**
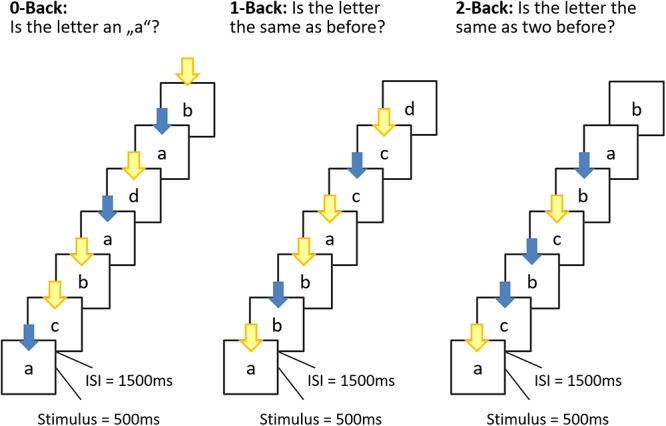
Schematic overview of the n-back task. Stimulus presentation was 500 ms, interstimulus interval (ISI) 1500 ms. During the ISI, a fixation cross was displayed. Participants were allowed to respond until the next stimulus appeared. In the 0-back condition, participants responded to the single pre-specified target letter “a”; in the 1-back condition, the target was any letter identical to the one immediately preceding it (i.e., one trial back); and in the 2-back condition, the target was any letter that was identical to the one presented two trials back. In this manner, the WM load (storage and manipulation demands) increased incrementally from the 0-back to the 2-back task. The color of the arrow refer to the button to be pressed (blue = match; yellow = mismatch).

Each trial consisted of a visual presentation of the letter a, b, c, or d (measuring 4 × 4 visual degrees; presented centrally on a monitor) for 500 ms followed by an interstimulus interval of 1500 ms. During a n-back block, participants had to indicate via key-press whether, or not, the stimulus of the current trial matched the stimulus they saw N-steps back (see Figure 1). One half of the trials of a block were matches, that is, required participants to press the blue marked key (“L”) as correct response, and one half of the trials were mismatches, that is, required participants to press the yellow marked key (“S”) as correct response. The sequences of matches and mismatches were pseudo-randomly generated with the constraint that after a maximum of three matches at least one mismatch followed. On a regular PC keyboard, the buttons “L” and “S” were used and participants were instructed to leave their index fingers on the two buttons for the duration of the task.

Each subject was given written and verbal instructions on the task. Before starting the test, participants completed a practice run (one block of each condition). Subjects were instructed to perform the tasks to the best of their ability, answering as quickly as possible without sacrificing accuracy by pressing the corresponding key. Each of the three conditions consisted of 36 trials, with a 60-s inter-condition rest period.

The main dependent variables were reaction time (ms) and accuracy (*A*^′^). The percentages of hits (correct match) and false positives (incorrect match) for the n-back task were used to calculate *A*^′^, a measure of detection sensitivity for each condition (Grier, 1971).

##### Flanker task

Inhibition was assessed by performance during a *modified flanker task* (Eriksen and Eriksen, 1974). The stimuli were presented using E-Prime software (Psychology Software Tools, Inc., Pittsburgh, PA) on a 17′′ computer monitor. Responses were registered using a standard QWERTZ keyboard. Participants sat approximately 70 cm away from the screen. During the task, participants attended to a centrally presented target stimulus (Chinese characters) amid an array of laterally presented flanking stimuli. During the compatible version of the task (all signs point in to the same direction: 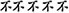), participants were required to press “L.” During the incompatible condition (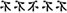), participants were required to press “S.” Five letter stimuli, measuring 4.5 cm tall and separated by 1 cm were presented for 750 ms on a white background. Participants were instructed to identify the central sign as quickly as possible without sacrificing accuracy by pressing the corresponding key. A randomized inter-stimulus interval of 400–1200 ms was used, and both the number of trials within each condition and the frequency of target direction were equiprobable, with randomly presented trials within each task block. Participants were administered five blocks of 16 trials for each compatibility condition and given a brief break and encouragement between each block. For all analyses, individual trials with RT’s outside the 200–1650 ms post-stimulus onset window and incorrect trials were excluded from the RT analysis (Wu et al., 2011). The main dependent variables were reaction time (ms) and accuracy (%) for congruent and incongruent trials.

#### Covariates

All participants were invited to a face-to-face interview to answer standardized questionnaires. The questionnaire included socio-demographic characteristics (i.e., age, gender, education level, marital status, dwelling space), lifestyle habits (i.e., physical activity, exercise; PAQ-50+; Huy and Schneider, 2008), depressive symptoms [Geriatric Depression Scale (GDS); Yesavage et al., 1983], and past medical history (e.g., diabetes).

##### General cognitive function

The MoCA (Nasreddine et al., 2005) was used to assess cognitive function, which is designed as a rapid screening tool for mild cognitive dysfunction. It assesses various cognitive domains, including attention and concentration, executive function, memory, language, visuo-constructional skills, conceptual thinking, and recall. Scores on the MoCa can range between 0 and 30, with lower scores indicating worse global cognitive functioning. The MoCA is a reliable and valid estimate of overall global cognitive abilities with a cutoff of 24 as optimal for a diagnosis of Mild Cognitive Impairment (Nasreddine et al., 2005; Kenny et al., 2013).

##### Depression

We applied the 15-item GDS (Yesavage et al., 1983), a self-report assessment to evaluate the degree of depressive symptoms of participants. The scores range from 0 to 15 points, with a higher score indicating a more depressive state. GDS scores of 0–5 were classified as having no depression, scores of 6–10 were classified as slight depression, and scores of 11–15 were classified as moderate to severe depression.

##### Physical activity and exercise

Everyday physical activity was assessed using the Physical Activity Questionnaire for the population aged 50 years and older (PAQ-50+, Huy and Schneider, 2008). The 37 questions are designed to estimate the physical activity of older adults based on different activities, including sport, housework, yard work, job, and leisure activities. Each activity can be evaluated by MET-values or the duration (min/week). The energy expenditure (kcal per week) can additionally be calculated by an equation from the specific activity/MET value, the duration of the activity performance and the weight of the person. The sum of all activities corresponds to the total result of the PAQ 50+ in kilocalories or the amount of time per week.

Subjects were also evaluated on their sports biography: participants were asked in which organized activities (participation through a formal club, max. three different activities) they had participated over the past 12 months (see also Schott et al., 2016). Next, they were asked how many days a week, and minutes per session, they had participated in that particular activity. Total exercise duration (h/week) was calculated as follows: (frequency 1 × duration 1) + (frequency 2 × duration 2) + (frequency 3 × duration 3).

##### Functional fitness

Physical performance was evaluated by two tests out of the Senior Fitness Test (SFT), which was developed for early identification of older adults who are at risk of losing functionality (Rikli and Jones, 2013): (1) 30s chair stand (assessment of lower body strength, repetitions within 30s), and (2) 30s arm curl (assessment of upper body strength, repetitions within 30s). The present study has followed the testing procedures suggested by the SFT manual. The 30s chair stand test consisted of recording the number of repetitions of rising from a chair and return to the seated position, as fast as possible, with their arms folded across their chest. The 30s arm curl consisted of recording the number of repetitions of biceps curls that can be completed in 30s, holding a hand weight of 2 kg for women and 3 kg for men.

##### Body composition

Height and weight were recorded using a standard protocol. Body mass index was calculated as weight in kilograms divided by height in meters squared (kg/m^2^).

### Procedure

Data was collected from May to September 2013. The questionnaires, the fitness and cognitive testing were administered individually during a single testing session lasting approximately 2 h, which included breaks as needed. All tests were administered by the same trained specialist (M.Sc. in Gerontology) with standardized procedures including standard instruction and practice. Additionally, the participants were encouraged to ask questions, if needed, throughout the procedures for better understanding and compliance.

### Statistical Analysis

Statistical analyses were implemented on SPSS v.25 (SPSS, Chicago, IL). We first explored dependent variables to examine missing data points, normality of distributions, and presence of outliers. The Kolmogorov–Smirnov tests indicated that all variables (except exercise, GDS, RTs) entered into the analysis were normally distributed.

Potential baseline group differences for continuous variables (i.e., age, height, weight, BMI, physical activity, education, fitness) were assessed using ANOVAs, and categorical demographic variables (i.e., gender) were compared by chi-square test.

Responses [accuracy (%, *A*^′^)] and reaction times of the n-back and/or flanker stimulus were recorded in every trial. Trials with reaction times longer or shorter than the median of all reaction times of a participant ±3 times the median absolute deviation were excluded from further analysis, as suggested by Leys et al. (2013). Median RTs for correct trials were then calculated for each condition as an indicator of processing efficiency. Flanker RTs and accuracy rates were submitted to ANCOVAs with repeated measures including the within-subjects factors cognitive load (block 1–5), congruency (congruent vs. incongruent) as well as the between-subjects factor of group (Master Athletes vs. Nuns/Monks vs. sedentary older adults) controlled for sex (due to the unequal distribution of men and women in our subsamples). n-back sensitivity (*A*^′^) and RT were compared similarly with cognitive load (0-back, 1-back, 2-back) as within-subjects factor and group as between-subjects factor controlled for sex. Analyses with three or more within-subjects levels report *p*-values after Greenhouse-Geisser correction for violations of sphericity. Significance levels were set at *p* = 0.05, and *post hoc* comparisons were conducted using Bonferroni correction. Partial eta-square ($\eta_{p}^{2}$) is reported to indicate effect size. Results of these analyses indicate whether, when inhibition and working memory were considered separately, there were significant differences on RT and error measures associated with group, cognitive load, and the interaction of group and cognitive load.

Pearson correlation analysis was employed to examine associations among all relevant variables. The independent variables with low explanatory power were not included in the regression analysis. Separate hierarchical stepwise regression analyses with orthogonal contrast coding were employed in order to examine whether differences between lifestyle groups remained when controlling for other relevant characteristics that might confound the association between lifestyle and cognitive functions. The first contrast compared Master Athletes and Nuns/Monks [-1] to the sedentary group [+2] (sed_active). The second contrast compared Master Athletes [-1] and Nuns/Monks [+1] (MA_NM). To address potential multicollinearity, the independent variables as well as the interaction terms were mean centered (Aiken and West, 1991). In the first block, the dependent variables from the n-back and the Flanker-task (accuracy values and RTs) were regressed on age, sex, and education. In block 2, the two group contrasts (sed_active, MA_NM), and in block 3, fitness, exercise, and physical activity were regressed on cognitive performance. Finally, in the fourth block, the corresponding product terms between group contrasts and fitness, exercise, and physical activity were entered.

## Results

### Participant Characteristics

A description of the three groups is given in Table 1. The three groups did not differ significantly in terms of age, years of education, general cognition (MoCA), and depression (GDS). The percentage of men and women was significantly different across the three groups. The proportion of male subjects was higher in the group of the Master Athletes than in groups of sedentary older adults (*p* = 0.057) and nuns and monks (*p* = 0.025). Additionally, Master Athletes had a lower BMI, took less medication, performed better on the 30s-Chair Rise test and the 30s-Arm Curl test, and exercised longer compared to the sedentary groups as well as the groups of nuns and monks.

**Table 1:** Participant characteristics by group.

	Sedentary (*n* = 20)	Nuns/Monks (*n* = 20)	Master Athletes (*n* = 20)	Statistical analysis
Age (years)	76.4 ± 5.96	77.5 ± 5.56	76.5 ± 5.33	*F*(2,57) = 0.23, *p* = 0.792, η^2^ = 0.008
Sex	6 M, 14 W	5 M, 15 W	12 M, 8 W	χ^2^(2) = 6.06, *p* = 0.048
BMI (kg/m^2^)	25.7 ± 3.02	26.3 ± 4.09	23.5 ± 3.32#	*F*(2,57) = 3.51, *p* = 0.036, η^2^ = 0.110
Medication (n)	0.75 ± 0.85	0.65 ± 0.75	0.15 ± 0.37*	*F*(2,57) = 4.39, *p* = 0.017, η^2^ = 0.133
Education (years)	11.1 ± 2.96	11.9 ± 3.57	11.8 ± 3.04	*F*(2,57) = 0.37, *p* = 0.692, η^2^ = 0.013
MoCA	27.5 ± 1.27	27.6 ± 1.02	27.5 ± 1.33	*F*(2,57) = 0.04, *p* = 0.963, η^2^ = 0.002
GDS	1.25 ± 1.68	1.25 ± 1.16	0.75 ± 0.64	*F*(2,57) = 1.09, *p* = 0.344, η^2^ = 0.037
30s-Chair Rise test (n)	15.4 ± 4.03	16.5 ± 4.54	22.0 ± 3.99###,***	*F*(2,57) = 14.2, *p* < 0.001, η^2^ = 0.333
30s-Arm Curl test (n)	18.8 ± 4.29	19.7 ± 4.02	28.0 ± 3.97###,***	*F*(2,57) = 30.5, *p* < 0.001, η^2^ = 0.517
PAQ (h/week)	19.0 ± 8.47	42.6 ± 14.3***	26.4 ± 16.7###	*F*(2,57) = 15.8, *p* < 0.001, η^2^ = 0.356
Exercise (min/week)	42.0 ± 64.1	37.5 ± 72.1	243.0 ± 109.8###,***	*F*(2,57) = 38.7, *p* < 0.001, η^2^ = 0.576

*Data are presented as mean ± standard deviation. Differences were evaluated by the Chi-square test or analysis of variance, followed by the Bonferroni test. BMI, body mass index; MoCA, Montreal Cognitive Assessment; GDS, Geriatric Depression Scale; PAQ, Physical Activity Questionnaire. ^∗^*p* < 0.05 and ^∗∗∗^*p* < 0.001 compared with the sedentary group. ^#^*p* < 0.05 and ^###^*p* < 0.001 compared with the nuns/monks*.

### Working Memory Performance

**Figure 2:**
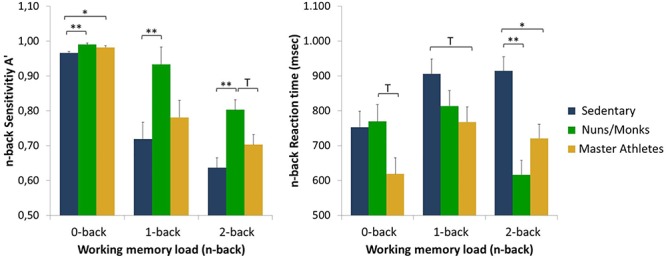
Detection sensitivity (A^′^) and reaction times as a function of working memory load (0-back, 1-back, and 2-back) and group (sedentary older adults, monks and nuns, master athletes). Bar graphs show sex adjusted mean ± SEM; ^∗^*p* < 0.05, ^∗∗^*p* < 0.01, and T *p* < 0.10.

Detection sensitivity scores (*A*^′^) and median reaction times for sedentary older adults, monks/nuns, and master athletes are presented in Figure 2. The manipulation of cognitive load was successful as indicated by a main effect of load, *F*(1.51,80.2) = 7.54, *p* < 0.001, $\eta_{p}^{2}$ = 0.125, with lower overall detection sensitivity in the 2-back (*M* = 0.714; SE = 0.016), than in the 1-back (*M* = 0.811; SE = 0.028), and the 0-back task (*M* = 0.979; SE = 0.003). The 3 (cognitive load) × 3 (group) interaction yielded also a significant effect, *F*(3.03,80.2) = 2.74, *p* = 0.048, $\eta_{p}^{2}$ = 0.094. *Post hoc* tests revealed that sensitivity was especially better in nuns/monks compared to sedentary older adults. In addition, there was a significant load × sex interaction, *F*(1.51,80.2) = 7.39, *p* = 0.002, $\eta_{p}^{2}$ = 0.122. Further analysis revealed no differences between men and women in the 0-back condition, but higher accuracy rates for women in the 1-back condition as well as higher accuracy rates for men in the 2-back condition.

The 3 (cognitive load) × 3 (group) repeated measures ANCOVA on median RTs controlled for sex revealed a load by group interaction, *F*(3.47,91.8) = 6.15, *p* < 0.001, $\eta_{p}^{2}$ = 0.188 with RTs slower for tasks with a higher load in the sedentary older adults, but faster RTs in the 2-back condition compared to the 1-back condition in nuns/monks, and master athletes. No main effects for cognitive load or the interaction load × sex were found.

### Inhibitory Control

**Figure 3:**
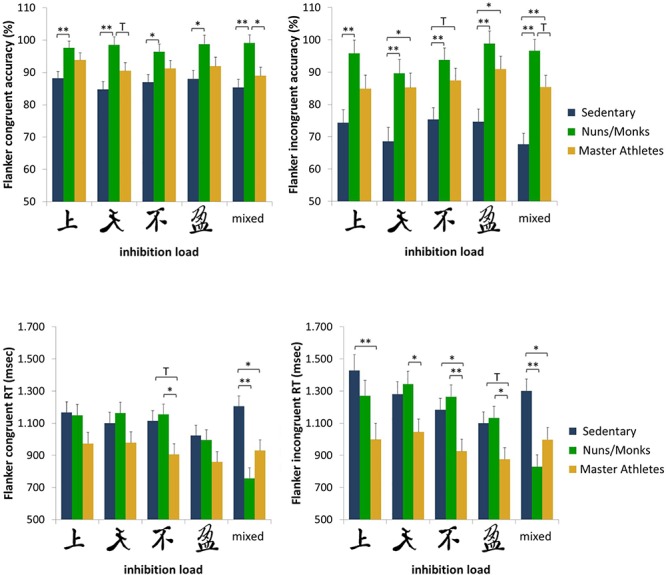
Accuracy rates and reaction times for congruent and incongruent trials as a function of inhibition load and group (sedentary older adults, monks and nuns, master athletes). Bar graphs show sex adjusted mean ± SEM; ^∗^*p* < 0.05, ^∗∗^*p* < 0.01, and T *p* < 0.10.

Accuracy scores and median reaction times for sedentary older adults, monks/nuns, and master athletes are presented in Figure 3. For the percentage of correct responses, the 5 (cognitive load) × 2 (congruency) × 3 (group) repeated measures ANCOVA controlled for sex resulted in a significant interaction effect of congruency × group, *F*(2,56) = 7.43; *p* = 0.001; $\eta_{p}^{2}$ = 0.210. *Post hoc* tests revealed the largest flanker interference for sedentary older adults (congruent 86.7% ± 1.77 vs. incongruent 72.1% ± 2.84) compared to the master athletes (congruent 91.3% ± 1.83 vs. incongruent 86.8% ± 2.93) and the nuns/monks (congruent 98.1% ± 1.79 vs. incongruent 94.9% ± 2.87). The main effects of load and congruency as well as the interactions load × group, load × sex, congruency × sex, load × congruency × group interactions were not significant.

To analyze performance of RTs in the flanker task, a 5 (cognitive load) × 2 (congruency) × 3 (group) repeated measures ANCOVA controlled for sex was conducted. Results revealed a main effect for congruency, *F*(1,56) = 8.39; *p* =0.005; $\eta_{p}^{2}$ = 0.130, showing faster RTs on congruent (1032 ms) than in incongruent trials (1132 ms). There was also a significant congruency × group interaction, *F*(2,56) = 5.48; *p* = 0.007; $\eta_{p}^{2}$ = 0.164. *Post hoc* tests revealed the largest flanker interference for sedentary older adults (congruent 1122 ms ± 56.7 vs. incongruent 1259 ms ± 64.5) compared to the nuns/monks (congruent 1044 ms ± 55.1 vs. incongruent 1168 ms ± 65.1) and the master athletes (congruent 929 ms ± 56.3 vs. incongruent 968 ms ± 66.5). The block × congruency × group interaction approached significance, *F*(6.32,177) = 1.86; *p* = 0.087; $\eta_{p}^{2}$ = 0.062 (see also Figure 3).

### Relationship Between Demographic Variables, Fitness, Physical Activity, and Cognitive Performance

Correlations among variables in the overall sample are reported in Table 2. Accuracy/sensitivity of working memory as well as inhibitory performance is positively associated with physical activity, while RTs are associated with age, sex, fitness, and exercise.

**Table 2:** Correlations between working memory and inhibition with demographic, fitness, and physical activity variables for all participants (*n* = 60).

	n-back A^′^ comp	n-back RT comp	Flanker congr ACC comp	Flanker incongr ACC comp	Flanker congr RT comp	Flanker incongr RT omp
Age (years)	0.16	0.32*	-0.24	-0.10	**0.32^∗^**	**0.28^∗^**
Sex	0.18	**0.30^∗^**	-0.03	-0.02	**0.33^∗^**	**0.29^∗^**
BMI (kg/m^2^)	-0.15	0.18	0.06	0.04	0.03	0.12
Medication (n)	-0.10	**0.33^∗^**	0.05	-0.07	0.17	0.22
Education (years)	0.19	-**0.28^∗^**	0.14	**0.27^∗^**	-0.19	-0.17
MoCA	-0.07	-0.14	0.05	0.08	-0.12	-0.20
GDS	0.14	0.13	-0.23	-0.14	0.17	0.19
30s-Chair Rise test (n)	-0.05	-**0.45^∗∗^**	0.07	0.10	-**0.45^∗∗^**	-**0.44^∗∗^**
30s-Arm Curl test (n)	-0.01	-**0.49^∗∗^**	0.18	0.23	-**0.52^∗∗^**	-**0.57^∗∗^**
PAQ (h/week)	**0.32^∗^**	-0.25	**0.37^∗∗^**	**0.48^∗∗^**	-0.11	-0.09
Exercise (min/week)	-0.09	-**0.31^∗^**	0.05	0.08	-**0.39^∗∗^**	-**0.40^∗∗^**

*Bolded values in black indicate statistically significant correlations (*p* < 0.05); comp, composite score; ACC, accuracy; congr, congruent; incongr, incongruent*.

Results for the hierarchical linear regression analyses are presented in Table 3. These analyses were conducted in order to examine if fitness, exercise, and physical activity explained a larger portion of variance than group (i.e., nuns/monks vs. Master Athletes vs. sedentary controls) in measures of working memory and inhibition irrespective of age, sex, and education. The contrast sed_active, which reflected our hypothesized cognitive performance pattern (sedentary controls < nons/monks and master athletes) reached significance in all variables, but the RTs for the congruent Flanker task. The alternate orthogonal contrast MA_NM reached significance for the composite sensitivity score for n-back, the accuracy rates for the congruent Flanker task as well as the RTs for the incongruent Flanker task, indicating better performance in accuracy measures in nuns/monks compared to master athletes. However, master athletes outperformed nuns/monks in RTs. Only for the RTs in the congruent condition of the Flanker task could we show an additive effect of fitness with higher-fit individuals showing faster RTs than low-fit individuals.

**Table 3:** Summary of separate hierarchical linear regression analysis for the interaction between demographic variables, orthogonal contrasts, fitness, exercise, and physical activity for the composite scores of the cognitive performance (only results for final models are presented).

Dependent variable	Predictor	*B*	SE *B*	β	*ΔR*^2^	*R*^2^
n-back A^′^	sed_active	-0.77	0.173	-0.490**	0.240	0.279
	MA_NM	0.68	0.299	0.251*	0.063	
n-back RT	Age	0.14	0.04	0.344**	0.102	0.353
	Sex	1.15	0.50	0.245*	0.089	
	sed_active	0.72	0.17	0.445**	0.195	
Flanker congr ACC	sed_active	-1.18	0.31	-0.435**	0.190	0.247
	MA_NM	1.35	0.53	0.287*	0.082	
Flanker in-congr ACC	Education	0.25	0.13	0.202*	0.071	0.385
	sed_active	-1.55	0.29	-0.552**	0.301	
	MA_NM	1.02	0.50	0.211*	0.044	
Flanker congr RT	Sex	1.47	1.09	0.173	0.108	0.276
	Age	0.17	0.09	0.224T	0.106	
	sed_active	0.39	0.37	0.132	0.062	
	Fitness	-0.13	0.06	-0.309*	0.050	
Flanker in-congr RT	Sex	1.53	1.00	0.183	0.082	0.242
	Age	0.21	0.08	0.279*	0.080	
	sed_active	0.85	0.33	0.294*	0.080	
	MA_NM	1.19	0.60	0.239*	0.052	

*Sed_active: Nuns/Monks/Master Athletes (coded as -1) versus sedentary controls (coded as 2); MA_NM: Master Athletes (coded as -1) versus Nons/Monks (coded as 1) vs. sedentary controls (coded as 0); ^*T*^*p* < 0.10, ^∗^*p* < 0.05, ^∗∗^*p* < 0.01; ACC, accuracy; congr, congruent; incongr, incongruent*.

## Discussion

A significant amount of studies has shown that there is an association between cognitive performance and lifestyle behaviors such as physical activity, fitness, sleep, nutrition, and spirituality in older adults (Clare et al., 2017). However, these studies usually included individuals who were not particularly inclined to exhibit these behaviors at all or were shown to adhere rather irregularly. Here, we report for the first time the relationship of stable, long-term lifestyles as can be found for Master Athletes and nuns/monks with cognitive performances. In the present study, we find that nuns/monks outperform Master Athletes as well as sedentary controls in accuracy values of working memory and inhibitory performance, while Master Athletes show faster reaction times compared to the other two groups. In addition, we hypothesized that higher levels of physical activity, exercise, and fitness would be associated with better cognitive performance. The data from our study partially supports this expectation. We found that fitness (strength of upper and lower extremities) and exercise were associated with faster reaction times (but not accuracy rates) on the n-back- and the Flanker Task. This indicates that older adults with a higher overall strength and longer durations of structured exercise produced faster reaction times. In contrast, greater physical activity levels were only correlated with accuracy rates of the n-back- and the Flanker Task.

### Determinants of Health Status in Nuns/Monks, Master Athletes, and Sedentary Controls

Elevated BMI may be associated with higher risk of dementia (Boyle et al., 2017). When compared to the Schienkiewitz et al. (2017) BMI data, it was apparent that Master Athletes (75%) and sedentary controls (50%), but not nuns/monks (40%) have a higher proportion of individuals with a BMI indicative of underweight or normal compared to the general German population in this age group (40%). Such results were similar with previous research that demonstrated a healthy BMI in Master Athletes (Fien et al., 2017) and nuns/monks (Bennett et al., 2018). Another essential aspect of preserving health and function in older adults is the ability to maintain muscle mass and power. All of the Master Athletes, 75% of the nuns/monks, and 60% of the sedentary controls in our study performed above criterion-referenced fitness standard for the 30s Arm Curl test and the 30s Chair Rise test (Rikli and Jones, 2013). Inspection of other health-related indicated that only 1.7% of our sample took three medications per day, and 3.3% had a GDS score of 5. 40% of the sedentary controls and 30% of the nuns/monks are engaged in exercise once or twice a week for at least 30 min; Master Athletes exercise twice (45%), three (35%) or five times (20%) a week for at least 60 min, which is similar to data reported by Fien et al. (2017). Eighty-five percent of the group of nuns/monks report daily leisure or gardening activities for at least 30 min each day; Master Athletes as well as sedentary controls are less active with 65%, respectively 50%. These moderate to vigorous levels of exercise and physical activities are also related to mortality: As shown in a previous study, Polish Olympic athletes experienced a slower rate of aging, a lower risk of mortality as well as a longer life-expectancy compared to nuns/monks and actors (Gajewski and Poznańska, 2008). Overall, our data suggests that we examined a functional and healthy group of older adults (Ravaglia et al., 2008).

### Cognitive Performance

As expected and in accordance with past research, that have demonstrated that older adults with higher fitness levels show better performances specifically in the executive domains (Taran et al., 2013; Thomas et al., 2013; Tseng et al., 2013; Alfini et al., 2016; Zhao et al., 2016; Dupuy et al., 2018), the Master Athletes in our study outperformed sedentary controls in both working memory performance as well inhibitory control (for reaction times, but not for accuracy). Our results are in line with Dupuy et al. (2018), who found in high-fit older adults faster reaction times for a Stroop task as a measure of inhibitory control. Similarly, our results align with Zhao et al. (2016) who reported better performances on a verbal memory task and a reaction time test [both from the Immediate Post-concussion Assessment and Cognitive Testing (ImPACT) tool] in Master Athletes compared to sedentary control. Also, Taran et al. (2013) showed significantly better performance on verbal learning and memory tasks (Rey Auditory Verbal Learning Test) as well as faster processing speed (Trail Making Test). In contrast, Wouters et al. (2017) found a positive relationship between gait speed as marker of fitness and working memory, but were not able to confirm a positive association between lifelong physical activity and working memory or executive function. Competitive cognitive engagement may be associated with better structural and functional connectivity across the human brain as expertise and the training of motor skills are often associated with changes in brain structure and function (Dayan and Cohen, 2011; Binder et al., 2017; Godde and Voelcker-Rehage, 2017; Gheysen et al., 2018). Indeed, Binder et al. (2017) found that a multi-domain motor-cognitive training improves neural lateralization and functional connectivity in older adults. Also, Tseng et al. (2013) reported that life-long exercise of Master Athletes resulted in higher gray and white matter concentrations in the subgyral, cuneus, and precuneus regions compared to sedentary controls; area which are related to visuospatial function, motor control, and working memory.

The group of nuns and monks represent a group of relatively homogenous individuals (e.g., income, occupation, and religiosity) with similar physical and socioeconomic characteristics of their communities. The rules of order life determine many health behaviors, which might be an effective lifestyle to increase brain- and cognitive-reserve capacity (e.g., low alcohol and tobacco consumption, daily prayer and meditation; Bowen and Luy, 2018). In general, nuns/monks outperformed Master Athletes and sedentary controls in both working memory performance as well inhibitory control (for accuracy) consistent with various attention related advantages that have been documented in older adults with meditation training (see for an excellent overview Christie et al., 2017). van Leeuwen et al. (2012) argue that meditation (similar to praying) improves the allocation of attention in space and the ability to adjust the focus of attention from a global pattern to the fine grained detail of an image (here Chinese characters). However, one has to take into account that meditation addresses the “self,” while spiritual practices focus on internal and external sense of connection to a higher entity (Barnby et al., 2015). To the best of our knowledge, studies comparing executive function of nuns and monks with community-dwelling older adults are non-existent. Larger studies as the “Religious Order Study” or the “Nuns Study” focuses on the examination of Alzheimer Disease pathology. In a recent attempt to describe a neurophysiological model of religious experiences, Newberg (2017) suggests a number of complex interactions of the prefrontal cortex (sustained attention) with the thalamus (alteration in the sense of realness and clarity), posterior superior parietal lobe (overall sense of self), limbic system (modulation or cortical arousal), and autonomic nervous system (relaxation, quiescence). In addition, he highlights the role of neurotransmitters (e.g., dopamine, serotonin, and acetylcholine). Since the frontal lobe is one of the brain regions most affected by aging processes, this shows the potential of meditative exercises in maintaining cognitive health in older adults (Christie et al., 2017).

Studies on the relationship of lifestyle factors and cognition have promoted the development of theoretical models that assess and attempt to identify the different biological, neurological, and psychological pathways by which e.g., fitness, exercise, intellectual engagement, meditation, sleep, and diet improve cognitive performance (especially cognitive control) such as the STAC-r (Reuter-Lorenz and Park, 2014), Cognitive Reserve Stern (2002), or the Adaptive Capacity Model (Raichlen and Alexander, 2017). The potential mechanisms for improved cognitive performance can be divided into three categories: neurobiological (e.g., neurotrophin gene; gray matter volume and activation; upregulation of neurotransmitters); psycho-social (e.g., self-efficacy, social connectedness, mood and emotions), and behavioral (e.g., quality of sleep, coping, self-regulation skills). For example, Raichlen and Alexander (2017) propose in accordance with the neurobiological hypothesis that energy saving mechanisms in response to reduced physical and cognitive activity would affect neurogenesis and synaptogenesis leading to reduced gray matter and regional brain atrophy. However, tolerance to neuropathological changes is attributed not only to neurobiological but also to psycho-social factors (Arenaza-Urquijo et al., 2015; Christie et al., 2017; Wilson and Bennett, 2017). Several studies found, that frequent participation in social activities are related with better cognitive performance, but especially emotional support and self-efficacy (beliefs about the ability to complete a task or achieve a goal) may be stronger associated with cognition than other psycho-social factors (Zahodne et al., 2014). Master Athletes exhibit not only higher levels of self-efficacy (Dionigi et al., 2011), but are also considered as exemplars of high social functioning (Geard et al., 2017).

Although our study has revealed intriguing novel findings, several limitations are worthy of note. First, we cannot conclude a causal link between lifestyle stability and cognitive performance, as this would require a longitudinal design. Indeed, the level of cognitive ability might influence the maintenance of physical activity or the cessation of smoking, as much as these habits might influence cognitive performance. In addition, due to our design, we cannot make any assumptions about cognitive function over time, since other genetic or environmental factors as well as other lifestyle behaviors (e.g., diet, smoking, alcohol consumption) may be involved, which were not included in our study (e.g., dietary and sleep patterns, smoking, alcohol consumption). Lifestyle behaviors, such as physical activity were self-reported, which may have led to recall bias. This could have underestimated the relationship between cognition and physical activity. Another limitation of the current study involves the lack of additional fitness measures (beyond strength tests), such as aerobic fitness (VO_2_max). Lastly, this study was based on a small, but unique sample size. Thus, the results must be interpreted with caution. However, the rules of a monastery as well as the rules of the successful participation in a competition require the constant observance of certain health-related behavioral patterns (Geard et al., 2017; Bowen and Luy, 2018).

## Conclusion

The primary novel results from this cross-sectional study with 20 nuns/monks, 20 Master Athletes, and 20 sedentary controls were that first, nuns/monks demonstrated better accurate cognitive performance than Master Athletes, while Master Athletes exhibited faster responses than both other groups. Second, fitness was associated with inhibitory control, and physical activity with the most demanding working memory task. Third, group was a statistically significant predictor for accurate performance, but not for reaction times. Our results on lifetime engagement in physical activity (Master Athletes) as well as “meditative exercises” (prayers, nuns/monks) seem to support the efficacy of stable and healthy lifestyles in preserving cognitive functioning. Although, we still have to speculate about why there is variance we cannot account for between our three groups (Lövdén et al., 2017). In the future, a key objective is to identify and describe the complex interaction of meaningful genetic, biological and environmental predictors, especially as they distinguish between exceptional groups and normal or groups with motor and/or cognitive impairment (Dixon and Lachman, 2019). Longitudinal studies are recommended to further examine the causal relationship of lifestyle stability and cognitive function in such specific cohorts.

## Ethics Statement

All participants were informed of the nature and aim of the study, and signed a consent form. All procedures were in accordance to the Declaration of Helsinki with ethical standards, legal requirements and international norms. An internal ethics committee at the University of Stuttgart approved the study.

## Author Contributions

NS conceived the experiment and analyzed the data. KK collected the data. NS and KK wrote the manuscript.

## Conflict of Interest Statement

The authors declare that the research was conducted in the absence of any commercial or financial relationships that could be construed as a potential conflict of interest.

## Footnotes

1 *Physical activity* is defined as any bodily movement produced by muscle action that increases energy expenditure. *Physical exercise* implies planned, structured, repetitive and purposeful physical activity, often with the goal of improving or maintaining one’s physical fitness. *Physical fitness* is a physiological state that provides the ability to carry out activities of daily living with optimal performance, endurance, and strength (Caspersen et al., 1985).

2 http://www.pstnet.com/eprime.cfm
